# Admission of Patients With Obstructive Sleep Apnea Undergoing Ambulatory Surgery in Otolaryngology—Head and Neck Surgery

**DOI:** 10.1177/00034894211048783

**Published:** 2021-09-30

**Authors:** Vincent Wu, Nick Lo, R. Jun Lin, Molly Zirkle, Jennifer Anderson, John M. Lee

**Affiliations:** 1 Department of Otolaryngology—Head and Neck Surgery, St. Michael’s Hospital, University of Toronto, Toronto, ON, Canada; 2 Department of Anesthesiology and Pain Medicine, St. Michael’s Hospital, University of Toronto, Toronto, ON, Canada; 3 Li Ka Shing Knowledge Institute, St. Michael’s Hospital, Toronto, ON, Canada

**Keywords:** obstructive sleep apnea, peri-operative, admission, Otolaryngology, surgery

## Abstract

**Objectives:**

Within Otolaryngology—Head and Neck Surgery (OHNS), obstructive sleep apnea (OSA) patients are frequently encountered. To implement policies and screening measures for admission of OSA patients undergoing ambulatory surgery, actual rates of admission must first be determined. We aimed to evaluate rates and reasons for admission of OSA patients after ambulatory OHNS surgery.

**Methods:**

Retrospective chart review was undertaken of all OSA patients undergoing elective day-surgery OHNS procedures at a tertiary center from January 1, 2018 to December 31, 2019. The primary outcome measure was percentage of OSA patients admitted to hospital after ambulatory OHNS surgery. Secondary outcome measures included reasons for admission. American Society of Anesthesiologists (ASA) score, perioperative complications, and patient demographics were captured.

**Results:**

There were 118 OSA patients, out of 1942 cases performed during the review period. Thirty-eight were excluded as the procedures were not considered ambulatory. The remaining 80 OSA patients were included for analysis, with an average age of 51.7, SD 13.8, and 30 (38%) females. The admission rate was 47.5% (38/80 patients). Admitted patients were older (*P* = .0061), and had higher ASA (*P* = .039). Indication for surgery or type of surgery did not differ among admitted and non-admitted patients. The majority of patients, 97% (37/38 patients), were admitted for post-operative monitoring.

**Conclusion:**

More than half of OSA patients did not require admission to hospital after ambulatory OHNS surgery, unaffected by indications for surgery or type of surgery. Higher ASA score and older age were found in admitted as compared to non-admitted patients.

## Introduction

Obstructive sleep apnea (OSA) is characterized by intermittent and recurrent episodes of partial or complete obstruction of the upper airway during sleep, associated with cessation of breathing. These episodes cause fragmented sleep and contributes to daytime sleepiness. OSA has been shown to be associated increased risk and rates of hypertension, stroke, congestive heart failure, diabetes mellitus, and all-cause mortality.^1-7^ Practice guidelines from the American Anesthesiology Association (ASA) have recommended longer post-operative monitoring of OSA patients after ambulatory surgery given the increased respiratory risk for desaturation.^8^ However, there exists institutional variability with regards to how these recommendations are implemented.

Policies surrounding the monitoring of OSA patients after surgery can vary across institutions from prolonged monitoring within the post-anesthetic care unit (PACU), to mandatory admission to a surgical ward. The department of Otolaryngology—Head and Neck Surgery at St. Michael’s Hospital in Toronto, Canada provides tertiary care primarily in sinonasal, laryngological, and endocrine surgeries. Current policies at our institution requires that all OSA patients be admitted, for extended monitoring, whereby a bed is specifically blocked off for the individual patient. Patients are admitted to the surgical ward from the PACU. Even when medically stable, patients with OSA cannot be discharged from the PACU. Based on our clinical experiences, a majority of the post-operative OSA patients do not require admission to hospital, and are actually discharged on the same day from the ward. Therefore, we estimate that there exist reducible healthcare costs as a result of current policies in the admission of all patients with OSA after ambulatory surgery.

In order to implement policies and screening measures for admission of OSA patients undergoing ambulatory surgery, actual rates of admission must first be determined. Herein, we aimed to evaluate the percentage of OSA patients requiring admission to hospital after ambulatory surgery in OHNS, and reasons for admission within our institution.

## Methods

### Ethics

This retrospective chart review was approved by the St. Michael’s Hospital research ethics board (#20-037c).

### Patient Selection

All patients with a history of diagnosed OSA, undergoing surgery within the OHNS department at St. Michael’s Hospitals, Toronto, Canada from January 1, 2018 to December 31, 2019 were included. Patients were excluded if they did not have ambulatory (outpatient) surgery. Moreover, patients with previously expressed wishes not to participate in research projects were excluded. The patient list was generated via the perioperative services. Data extraction was performed from pre-existing electronic medical charts.

### Outcome Measures

The primary outcome measure was percentage of OSA patients admitted to hospital. Secondary outcome measures included reasons for admission based on the discharge summary. Additionally, type of surgical procedure, indication for surgery, ASA score of the patient for the listed surgery, presence of any intraoperative (ie, desaturation, difficult intubation) or post-operative events (ie, desaturations, increased oxygen requirement), length of stay (LOS) in hospital, and patient demographic were captured.

### Statistical Analysis

Data was inputted into a spreadsheet designed specifically for the project. Descriptive statistics were used to highlight the percentage of admitted patients, baseline demographics, and reasons for admission. Comparison of categorical variables between admitted and non-admitted patients was performed using Chi-squared/Fisher’s exact test, and comparison of mean difference was performed with Student’s *t*-test given normally distributed data. Multinomial logistic regression was used to determine whether age, gender and ASA score were independent predictors for post-operative admission. All statistical analyses were performed with SPSS (v.25, Internal Business Machine, United States), and statistical significance was set to *P* < .05.

## Results

There were 1942 OHNS surgical cases performed during the 2-year review period. Of these, 118 patients had a diagnosis of OSA, which represented 6.1% of total cases. Thirty-eight patients were excluded as the procedures were not considered ambulatory in nature (ie, total thyroidectomy, neck dissection). The remaining 80 OSA patients were included for analysis. Patient demographics are shown in Table 1.

**Table 1.** Patient Demographics.

Characteristics	Admitted (n = 38)	Non-admitted (n = 42)	Total (n = 80)	*P*-value
Age (mean, SD)	56.3, 13.5	47.4, 14.7	51.7, 13.8	<.01
Female (n, %)	16, 53	14, 47	30, 38	.56
ASA (mean, SD)	3.03, 0.49	2.76, 0.62	2.89, 0.57	<.01

For the 80 OSA patients who had ambulatory OHNS surgery, 38 were admitted, with an admission rate of 47.5%. Age of the patient (*P* = .027) and ASA score (*P* = .007) were both independent predictors of post-operative admission. Patients admitted were significantly older as compared to non-admitted patients (mean difference 8.9 years, 95% CI, *P* = .0061). ASA scores was significantly higher in admitted patients as compared to non-admitted (mean difference, 95% CI *P* = .039). Gender was not significantly different between admitted and non-admitted patients (*P* = .49). Surgical procedures were also evaluated, and categorized based on OHNS subspecialties (Table 2). Differences in rate of admission were not statistically significant between surgical procedures (*P* = .19).

**Table 2.** Breakdown of Procedures by Sub-Specialty.

Sub-specialty	Admitted (n = 38)	Non-admitted (n = 42)	Total (n = 80)
Rhinology (n, %)	32, 53	28, 47	60, 75
Laryngology (n, %)	4, 31	9, 69	13, 16
General OHNS (n, %)	2, 29	5, 71	7, 8.8

Operative notes were reviewed for indications for surgery and included as part of Table 3. Specifically, there were 6 patients with a diagnosis of OSA as the indication for surgery. All 6 underwent tonsillectomy, and 2 (33%) were admitted for overnight observation. There were no statistical differences between admission rates based on pre-operative diagnoses (*P* = .31). No intra-operative events or complications were reported. There was 1 patient, who post-operatively had hypercapnic respiratory failure and serotonin syndrome requiring intensive care unit (ICU) admission, due to a pre-existing medical condition. No other post-operative complications were noted.

**Table 3.** Pre-Operative Diagnosis for Surgery.

Pre-operative diagnosis	Admitted (n = 38)	Non-admitted (n = 42)	Total (n = 80)
Chronic rhinosinusitis (n, %)	21, 57	16, 43	37, 46
Nasal obstruction (n, %)	11, 48	12, 52	23, 29
OSA (n, %)	2, 33	4, 67	6, 7.5
Laryngeal pathology (n, %)	4, 31	9, 69	13, 16
Other (hearing loss) (n, %)	0, 0	1, 100	1, 1.3

Patients’ discharge summaries were evaluated for reasons for admission and post-operative complications. The majority of patients, 37/38 (97%), were admitted for “monitoring.” The average LOS was 1.07 days (standard deviation 0.48 days); 37/38 (97%) patients had a LOS of 1 day, aside from 1 case requiring ICU admission who had LOS of 4 days.

## Discussion

Through this retrospective review, we found that more than half of OSA patients (52.5%) did not require admission to hospital for monitoring of their OSA after ambulatory OHNS surgery, unaffected by their indications for surgery. In admitted OSA patients, we found that there was higher ASA score and older age, as compared to those not admitted. Kandasamy et al^9^ evaluated 345 consecutive patients with OSA undergoing UPPP, and noted an overall admission rate of 71.9%. This is more than the reported admission rates from our review. However, we evaluated all OHNS ambulatory surgeries and not only UPPP. It should be noted that within OHNS, there exists significant differences between surgeries performed for the management of OSA, which are often within the oropharynx, as compared to other sinonasal, otologic, or minor head and neck procedures.^10^ The implications of operations performed within the pharynx have the theoretic risks of increased airway edema, upper airway obstruction, or bleeding into the airway.

Nevertheless, Kandasamy et al^9^ noted the incidence of complications among OSA patients to be low (12.8%), with suggestions that in select patients, outpatient UPPP would be safe. The authors further suggested that those patients with more severe OSA with increased apnea-hypopnea index, higher body mass index (BMI), or multiple comorbidities are at higher risk for postoperative complications and are most appropriate for overnight monitoring. Additionally, Liao et al^11^ evaluated risk factors for post-operative complications between OSA and non-OSA patients, finding that OSA patients tended to have increased rates of obesity and medical co-morbidities. Therefore, it appears that within OSA patients, more significant co-morbidities, including increased BMI, is associated with increased risk for post-operative complications, and should be a factor in the decision for admission and monitoring.

A vast majority of the patients in our study had no complications during their admission, and were admitted for overnight monitoring. Of the 47.5% who were admitted, all but 1 patient had a LOS of 1 day, without complications, suggesting that risk stratification may be performed to further decrease admission rates. Prevention of perioperative complications in patients have been studied, including the introduction of screening questionnaire. Checklists such as STOP-BANG serves as a possible indicator of OSA severity, and additionally helps to screen those who may have undiagnosed OSA.^12^ Furthermore, Baugh et al^13^ evaluated 452 OSA patients undergoing surgery and found that 89% of the procedures were performed on an ambulatory basis, and that there were no catastrophic complications. Baugh et al^13^ concluded that ambulatory surgery treating OSA may be performed safely for many patients on an outpatient basis. The Society for Ambulatory Anesthesia, after review of the current evidence, issued a revised algorithm for perioperative management of patients with OSA, different than that of the ASA, concluding that patients with mild-to-moderate OSA and appropriately managed comorbidities could safely undergo ambulatory surgery.^14,15^ Together, evidence suggests that with risk stratification, ambulatory surgery can be safely performed in select OSA patients.

Acknowledging that there exists higher risk of post-operative complications in patients with OSA, mainly with regards to oxygen desaturation events, the main objectives of this research was to evaluate a broad based policy centered on mandatory admission of all patients. Policies such as these ultimately are centered around improving patient care, such that if needed, a bed is blocked off and always available for OSA patients after ambulatory surgery. Some considerations with such a policy is that it does not allow for risk stratification of patients based on the severity of their OSA or presence of other comorbidities. Furthermore, the utilization of an OSA diagnosis as a screening tool may be overly cautious of those that may experience a post-operative respiratory event, especially if the OSA patient is treatment compliant. With regards to healthcare associated costs, according to data from the Canadian Institute for Health Information,^16^ the average cost of a standard hospital stay ranged from $4900 to $10 000 Canadian dollars. All in all, policies such as these have the potential to increase healthcare associated costs, and contribute to increased administrative burdens on physicians and allied healthcare professionals.

There were potential limitations to this study. Firstly, this was a retrospective study, and as such there exist the possibility for selection bias. While we aimed to cross-check the list of OSA patients who had ambulatory OHNS surgery provided by perioperative services with surgeons’ individual operating lists, there exists the chance that individuals with OSA could have been missed. As well, this methodology would not account for individuals who have undiagnosed OSA. Patients with undiagnosed OSA may be more challenging than those with treated OSA, as those with treated OSA, with positive pressure devices, are protected, as long as they wear their devices. Various screening tools including the STOP-BANG scoring system may provide additional metrics by which high risk patients are identified. Moreover, patient comorbidities, severity of OSA, positive pressure device adherence, and obesity were not captured. Despite the lack of data surrounding these measures, we still noted a significant number of patients either discharged the same day, or discharged after a LOS of 1 day. Future studies can aim to improve on these limitations, and provide a broader review amongst other surgical divisions across different academic and community hospital sites. Formal assessments can be undertaken to evaluate healthcare costs associated with broad based policies on admitting all OSA patients after ambulatory surgery.

## Conclusion

More than half of OSA patients, 52.5%, did not require admission to hospital for monitoring of their OSA after ambulatory OHNS surgery, unaffected by their indications for surgery, or type of surgery. Additionally, we found higher ASA score and older age in admitted as compared to non-admitted patients. Policies based on risk stratification should be used when considerations are made for admitting OSA patients after ambulatory surgery.
